# Supplementary material for: Nitrogen- and phosphorus-starved Triticum aestivum show distinct belowground microbiome profiles
Source: PLoS One. 2019 Feb 20;14(2):e0210538. doi: 10.1371/journal.pone.0210538 (PMC6382137; doi:10.1371/journal.pone.0210538)
Supplement: S1 Table — (DOCX) [file pone.0210538.s001.docx]

**S1 Table.** **Sample summary.** Summary of samples analyzed in this study.

| Sample type | Jun-2013 | Jul-2013 | Jun-2014 | Jul-2014 |
| --- | --- | --- | --- | --- |
| Roots | 16 (4 replicates per plot) | 16 (4 replicates per plot) | 16 (4 replicates per plot) | 15 (3 replicates in N0P20) |
| Rhizosphere | 0 | 16 (4 replicates per plot) | 15 (3 replicates in N0P20) | 15 (3 replicates in N0P20) |
| Bulk soil | 16 (4 replicates per plot) | 16 (4 replicates per plot) | 16 (4 replicates per plot) | 15 (3 replicates in N0P20) |
